# Growth in the Aftermath of Psychosis: Characterizing Post-traumatic Growth in Persons With First Episode Psychosis in Singapore

**DOI:** 10.3389/fpsyt.2021.784569

**Published:** 2022-01-26

**Authors:** Ying Ying Lee, Vanessa Seet, Yi Chian Chua, Swapna Kamal Verma, Mythily Subramaniam

**Affiliations:** ^1^Research Division, Institute of Mental Health, Singapore, Singapore; ^2^Early Psychosis Intervention Programme, Institute of Mental Health, Singapore, Singapore; ^3^Education Office, Duke-NUS Medical School, Singapore, Singapore; ^4^Saw Swee Hock School of Public Health, National University of Singapore, Singapore, Singapore

**Keywords:** schizophrenia, post-traumatic growth, first episode psychosis (FEP), growth, psychosis

## Abstract

Experiencing first episode psychosis (FEP) is a highly traumatic life event. However, there is evidence to show that the outcome of psychosis is more nuanced than was conventionally thought. Young persons with FEP can grow from the experience of psychosis. In this study, we aim to characterize post-traumatic growth (PTG) in persons with FEP over 1 year. A total of 99 FEP clients receiving services from an early psychosis intervention team in Singapore were recruited. The PTG Inventory, among other scales, like Questionnaire on the Process of Recovery and Connor-Davidson Resilience Scale, were administered in this population. A total of 52 participants completed the questionnaire at two timepoints (one year apart). The Reliable Change Index was calculated for participants who completed both timepoints. Repeated measures of correlation were performed, which identified personal recovery and resilience to be associated with PTG in this sample. This clinical population exhibited PTG in the aftermath of psychosis. PTG was associated with personal recovery and resilience, but not clinical indicators, like symptoms and functioning. Data from this study suggests that recovery and growth from first episode psychosis is a possibility. Clinical implications, strengths and limitations of this study are discussed.

## Introduction

Psychotic disorders are serious mental health conditions with great burden and poor outcomes (1, 2). However, the advent of early intervention for psychosis changed the illness trajectory for many young persons who experienced their first episode of psychosis (FEP) to one of hope and recovery (3). The road to recovery from psychosis is fraught with many challenges and obstacles for a young person. It is well-documented in the literature that experiencing first episode psychosis can result in post-traumatic stress disorder (4, 5). Evidence of decline in cognitive and social functioning in persons with psychosis has also been reported (6). Furthermore, these young people also face tremendous societal and internalized stigma (7, 8). Despite the odds being stacked against them, growth was still reported among those who experienced first episode psychosis across the globe (9–12).

Evidence of post-traumatic growth (PTG) in persons with first episode psychosis burgeoned in the last two decades. Since its initial qualitative characterization (9, 13, 14), several quantitative studies followed to further characterize PTG in persons with first episode psychosis in various settings (10, 11, 15). It was observed that the study participants' response to first episode psychosis was not binary (either distress or growth). Rather, it was a nuanced mix of both distress and growth (9, 10). Moreover, PTG was mediated by the respondents' ability to make sense of their suffering and adopt positive coping styles (10). Mazor et al. (10) highlighted the benefits of exploring and developing coping strategies among persons recovering from psychosis. It meant that growth was not just a possibility for persons living with psychosis but could be intentionally fostered in the clinical setting.

Most studies that delved into PTG of persons with first episode psychosis used the PTG Inventory (PTGI) to measure this phenomenon (16). PTGI was first developed by Tedeschi and Calhoun (17). Since its development in the 1990s, a number of confirmatory factor analysis studies were performed on PTGI to identify the dimensions of PTG (18, 19). Out of the models tested, there was consensus that the five-factor model had the best fit. The five domains were—relating to others, personal strength, new possibilities, spirituality, and appreciation for life.

Despite the amassed evidence of PTG in people with first episode psychosis internationally, no longitudinal study has been performed to track its development over time (12). Moreover, to the best of the authors' knowledge, there are no studies on the phenomenon of growth in the aftermath of psychosis in the Asian context. It is also not known if PTG is culturally relevant to people with FEP in Singapore. Moreover, critics of the PTGI postulated that a scale that measured perceived growth may be different from the actual growth that one may experience (20). They argued that the inability of respondents to accurately remember intrapersonal and interpersonal growth may affect the data collected from a scale that measures retrospective recall of PTG.

Hence, the aims of this study were to 1) see if there was convergence between the questionnaires that measured actual growth vs. perceived growth using PTGI, 2) characterize PTG in a multi-ethnic sample population of first episode psychosis patients in Singapore over 1 year, and 3) identify constructs associated with PTG in the same population.

## Methods

### Participants and Ethical Approval

Ethical approval was sought and received by the National Healthcare Group's Domain Specific Review Board (DSRB) (reference: 2018/01278). A total of 99 participants were recruited from the Early Psychosis Intervention Programme (EPIP) at the Institute of Mental Health, Singapore, from April 2018 to May 2021. EPIP is a comprehensive, integrated, patient-centered program led by a multidisciplinary team of psychiatrists, psychologists, case managers, social workers, nurses, occupational therapists, and peer support specialists. They specialize in working with youths and adults aged 16–40 years of age who experienced psychosis for the first time. The team provides both inpatient and outpatient care, supplemented by a wide range of psychosocial programs to promote recovery and reintegration with the society for these young people. Some specific interventions offered to EPIP patients include case management, psychotherapy, peer support, psychosocial groups, occupational therapy, and pharmacotherapy.

The inclusion criteria for the participants of this study included those who had experienced psychosis; and were 1) receiving treatment with EPIP for 10–14 months, 2) above 21 years old, 3) able to communicate in English, 4) able to complete a 30-min self-administered survey, and 5) able to give informed consent. Sociodemographic information like gender, age, ethnicity, occupation etc. were collected from the participants as well. Data were collected at two time points (Time 1 – T1 and Time 2 – T2) for a subgroup of participants to gather data over a period of 1 year. A total of 52 participants completed the surveys at both time points, giving a follow-up rate of 75% at the point when the project grant expired (May 2021). Clinical data from EPIP's standing database was also included. The data requested included duration of untreated psychosis (DUP), Positive and Negative Syndrome Scale (PANSS) scores (at baseline, 1 year and 2 years), and employment status (at baseline, 1 year and 2 years). More details of the self-administered scales are described below.

### Self-Administered Survey

#### Post-traumatic Growth Inventory

This is a 21-item survey that measures PTG (17). It measures five different domains of growth (relating to others; new possibilities; personal strength; spiritual change; appreciation of life) in the aftermath of trauma. Respondents answer each item on a 6-point Likert scale ranging from 0 (I did not experience this as a result of my crisis) to 5 (I experienced this change to a very great degree as a result of my crisis). Total scores range from 0 to 105. Higher scores are indicative of more growth after crisis. The use of this scale in our sample showed good internal consistency with Cronbach's α = 0.96 (T1), and α = 0.97 (T2).

#### Ryff's Psychological Well-Being Positive Relationship Subscale

This is a 7-item subscale that measured positive relationships with others developed by Ryff (21). It has a 6-point Likert scoring system (from strongly disagree to strongly agree). Total scores range from 6 to 42. Higher scores are indicative of better relationships with others. Example questions include “Most people see me as loving and affectionate.” “I find it difficult to really open up when I talk with others.” In this sample, this scale showed moderate internal consistency with Cronbach's α = 0.75 (T1), and α = 0.77 (T2).

#### Gratitude Questionnaire

It is a 6-item scale (22) that measures gratitude in its respondents. It is scored by a 7-point Likert-type scale from strongly disagree to strongly agree. The questionnaire total scores range from 6 to 42. Higher scores are indicative of stronger gratitude in respondents. Sample questions included “I have so much in life to be thankful for.” “I am grateful to a wide variety of people.” The use of this scale in our sample showed good internal consistency with Cronbach's α = 0.81 (T1), and α = 0.83 (T2).

#### Satisfaction With Life

It is for all a 5-item scale that measures the respondent's satisfaction with life (23). It has a 7-point Likert scoring, ranging from strongly disagree to strongly agree. Total scores range from 5 to 35. Higher scores are indicative of higher satisfaction with life. Sample questions are “In most ways, my life is close to my ideal.” “If I would live my life over, I would change almost nothing.” In this sample, this scale showed good internal consistency with a Cronbach's α = 0.89 (T1), and α = 0.91 (T2).

#### Meaning in Life Questionnaire

It is a 10-item scale (24) designed to measure two dimensions of meaning in life (presence of meaning and search for meaning). Respondents answer each item on a 7-point Likert-type scale ranging from 1 (Absolutely Untrue) to 7 (Absolutely True). Total scores range from 7 to 70. Higher scores are indicative of greater perceived meaning in life. Examples of items include “I understand my life's meaning.” “My life has no clear purpose.” The use of this scale in our sample showed good internal consistency with Cronbach's α = 0.81 (T1), and α = 0.83 (T2).

#### Religious Commitment Inventory-10

This is a 10-item scale that measures religious commitment of respondents (25). It has a 5-point Likert-type scale, ranging from not at all true of me to totally true of me. Total scores range from 10 to 50. Higher scores are indicative of higher religious commitment. Example questions include “I spend time to grow in my understanding of my faith.” “It is important for me to spend periods of time in private religious thought and reflection.” In this sample, this scale showed good internal consistency with Cronbach's α = 0.96 (T1 and T2).

The questionnaires on Ryff's positive relationship, meaning in life, satisfaction in life, gratitude, and religious commitment were administered in conjunction with PTGI for a parallel measure of PTG. This is because PTGI is a retrospective survey that requires respondents to 1) recall their prior experiences 2) assess their current experiences 3) gauge the change in their lives because of the crisis (20). Hence, it may not be the most accurate scale for the measure of actual PTG. Frazier et al. (20) proposed an alternative that is to measure the five domains assessed (relating to others, spirituality, new possibilities, appreciation of life and personal strength) in PTGI directly. For instance, Ryff's positive relationship with others is used to match the domain of relating to others; meaning and satisfaction of life to match appreciation of life; religious commitment inventory to match spirituality (20).

#### Connor-Davidson Resilience Scale

It is a 10-item scale that measures the resilience of its respondents (26, 27). It has a 5-point response scale ranging from 0 (not true at all) to 4 (true nearly all the time). The total score ranges from 0 to 100 with higher scores indicating higher resilience. Questions include “I can deal with whatever comes my way.” In this sample, this scale showed good internal consistency with Cronbach's α = 0.95 (T1), and α = 0.94 (T2).

#### Questionnaire About the Process of Recovery

It is a 15-item survey that measures the personal recovery of persons with psychosis (28). Each item of the scale is scored on a 5-point Likert scale ranging from 0 (strongly disagree) to 4 (agree strongly). Scores range from 0 to 60. Higher scores are indicative of recovery. Examples of items in survey include “I feel better about myself.” “My experiences have changed me for the better.” The use of this scale in our sample showed good internal consistency with a Cronbach's α of 0.95 (T1), and α = 0.77 (T2).

### Data Analysis

For the PTGI scale, responses were categorized as indicating “no to a small amount” of growth for individual item scores of 2 and below, and “moderate to great amount” of growth for scores of 3 and above (11). Reliable Change Indices [RCIs; (29)] were computed to assess clinically significant change in self-reported growth from Time 1 to Time 2, with an RCI of 1.96 and above indicating reliable increase in growth scores. Convergence of the PTGI scale with the five domains was measured using Pearsons' correlations. Finally, repeated measures correlations via the *rmcorr* package were used to examine associations between post-traumatic growth and personal recovery (QPR), resilience (CD-RISC-10), self-stigma (ISMI), psychotic symptom severity (PANSS), employment and duration of untreated psychosis (30). Repeated measures correlation is a statistical method to compute the average correlation between two variables at two or more time points (30). Due to the within-subject design of the study, to account for intra-individual associations in these measures taken at both timepoints, weighted correlation coefficients were computed via subject means and tested (31). All analyses were conducted with R, version 4.0.2 (32).

## Results

Table 1 shows the sociodemographic profile of the respondents in this study (n = 99). In the sample, there was an almost equal split of male and female respondents (female = 52.5%). The ethnicity profile is similar to that of the population in Singapore (33). Most respondents were single or never married. The bulk of the respondents had at least tertiary level education (diploma = 34.3% and degree = 32.3%). Most respondents were either employed (39.4%) or in between employment (32.3%). All participants were recipients of treatment at EPIP, with most of them living with the diagnosis of schizophrenia (29.3%).

**Table 1:** Sociodemographic profile of the participants.

***n* = 99**	***n***	**%**
**Gender**		
Female	52	52.5
Male	47	47.5
**Ethnicity**		
Chinese	69	69.7
Indian	10	10.1
Malay	15	15.2
Others	5	5.1
**Marital**		
Divorced/separated	2	2.0
Married	14	14.1
Single/never married	83	83.8
**Education**		
A Level/Pre-U	6	6.1
Degree	32	32.3
Diploma	34	34.3
O/N level	16	16.2
Post graduate degree (e.g., Masters/PhD)	3	3.0
Vocational certificate	6	6.1
Others (specify)	2	2.0
**Employment**		
Employed	39	39.4
In-between employment	32	32.3
Student/Homemaker	25	25.3
Others	3	3.0
**Diagnosis**		
Bipolar disorder with psychotic features	8	8.1
Brief psychotic disorder	11	11.1
Delusional disorder	4	4.0
Depression with psychotic features	9	9.1
Schizoaffective disorder	6	6.1
Schizophrenia	29	29.3
Schizophreniform	9	9.1
Others (specify)	13	13.1
I don't know/I don't want to share	9	9.1
Missing data	1	1.0

The PTGI performed well as a scale with good internal consistency (Cronbach's alpha = 0.96–0.97) and good convergence with relevant scales in our sample population. Table 2 shows the convergence of PTGI with relevant scales that measure “actual” change (20) in domains of relating to others, new possibilities, personal strength, spiritual change, and appreciation for life. The total PTGI scores correlated significantly with Ryff's positive relationship subscale (*r* = 0.27, *p* <0.01), satisfaction in life (*r* = 0.40, *p* < 0.001), meaning in life (*r* = 0.68, *p* < 0.001), religious commitment inventory (*r* = 0.41, *p* < 0.001) and gratitude questionnaire (*r* = 0.37, *p* < 0.001). When the specific domains of PTG were examined (relating to others, new possibilities, personal strength, spiritual change and appreciation for life), significant correlations were observed with matching scales like Ryff's positive relationship (*r* = 0.32, *p* < 0.01), satisfaction in life (*r* = 0.34, *p* < 0.001), meaning in life (*r* = 0.68, *p* < 0.001), religious commitment inventory (*r* = 0.56, *p* < 0.001), and gratitude questionnaire (*r* = 0.42, *p* < 0.001), respectively.

**Table 2:** Convergence of PTGI with various indicator scales.

**Pearson's r**	**Ryff's positive** **relationship**	**Satisfaction** **in life**	**Meaning** **in life**	**Religious commitment** **inventory**	**Gratitude** **questionnaire**
PTGI total	0.27^**^	0.40^***^	0.68^***^	0.41^***^	0.37^***^
PTGI (relating to others)	0.32^**^	0.32^**^	0.52^***^	0.24^*^	0.29^**^
PTGI (new possibilities)	0.18	0.34^***^	0.71^***^	0.40^***^	0.33^**^
PTGI (personal strength)	0.25^*^	0.42^***^	0.68^***^	0.40^***^	0.38^***^
PTGI (spiritual change)	0.23^*^	0.38^***^	0.57^***^	0.56^***^	0.23^*^
PTGI (appreciation for life)	0.16	0.35^***^	0.48^***^	0.37^***^	0.42^***^

* *p < 0.05;*

** *p < 0.01;*

*** *p < 0.001*.

**Figure 1:**
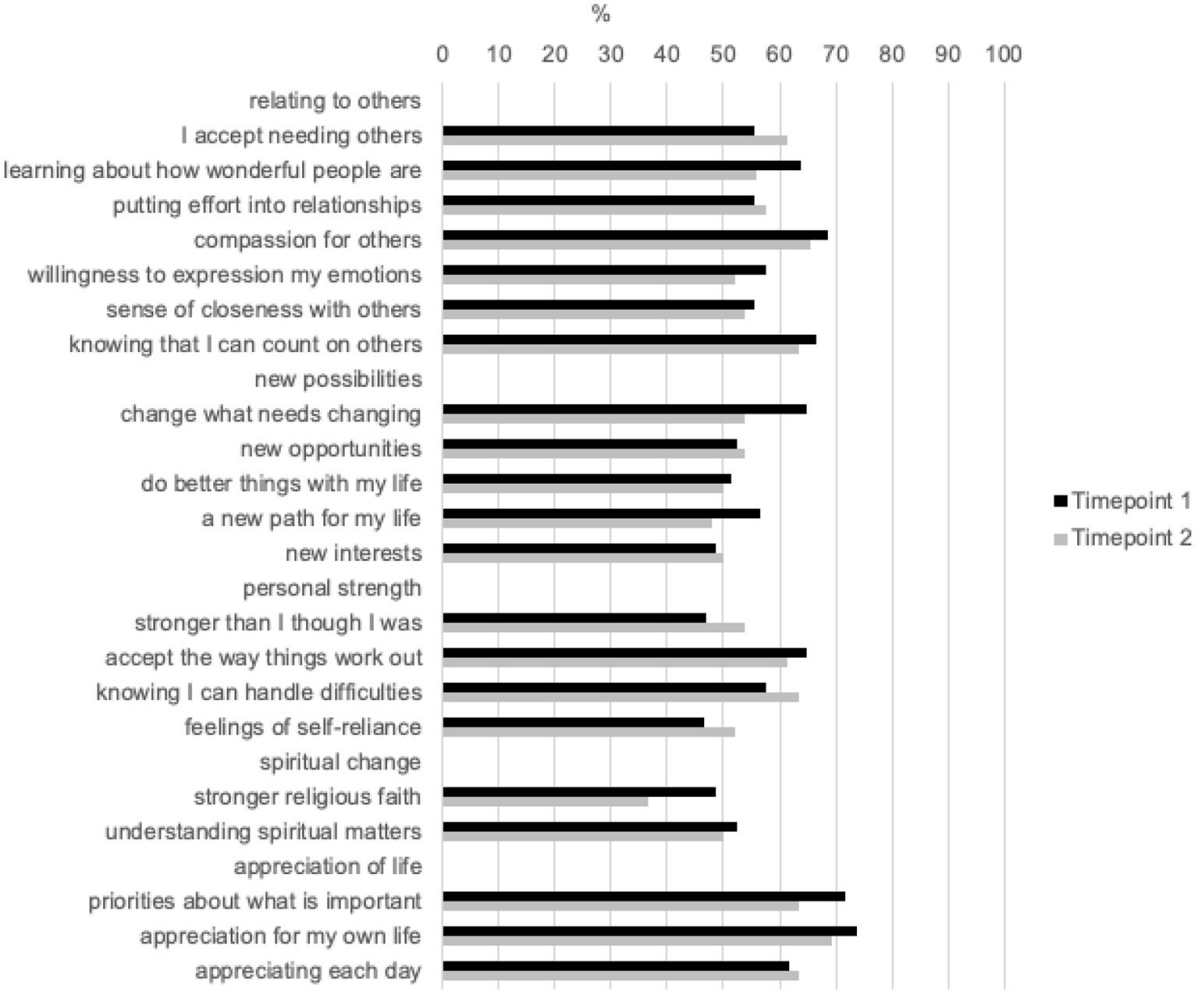
Participants' endorsement of moderate to very great change in PTGI.

Most of our respondents reported a moderate to very great change in the PTGI score in all domains of the PTGI scale. Figure 1 shows a breakdown of the individual questions from the PTGI scale and the rate of endorsement in percentage for moderate to very great change. Additionally, we examined data at T1 and T2. Out of the five domains in the PTGI scale, respondents reported the largest change in the domain of appreciation for life (T1 = 71.7%; T2 = 63.5%), while respondents reported lowest change in the domain of spiritual change (T1 = 48.5%; T2 = 36.5%).

**Figure 2:**
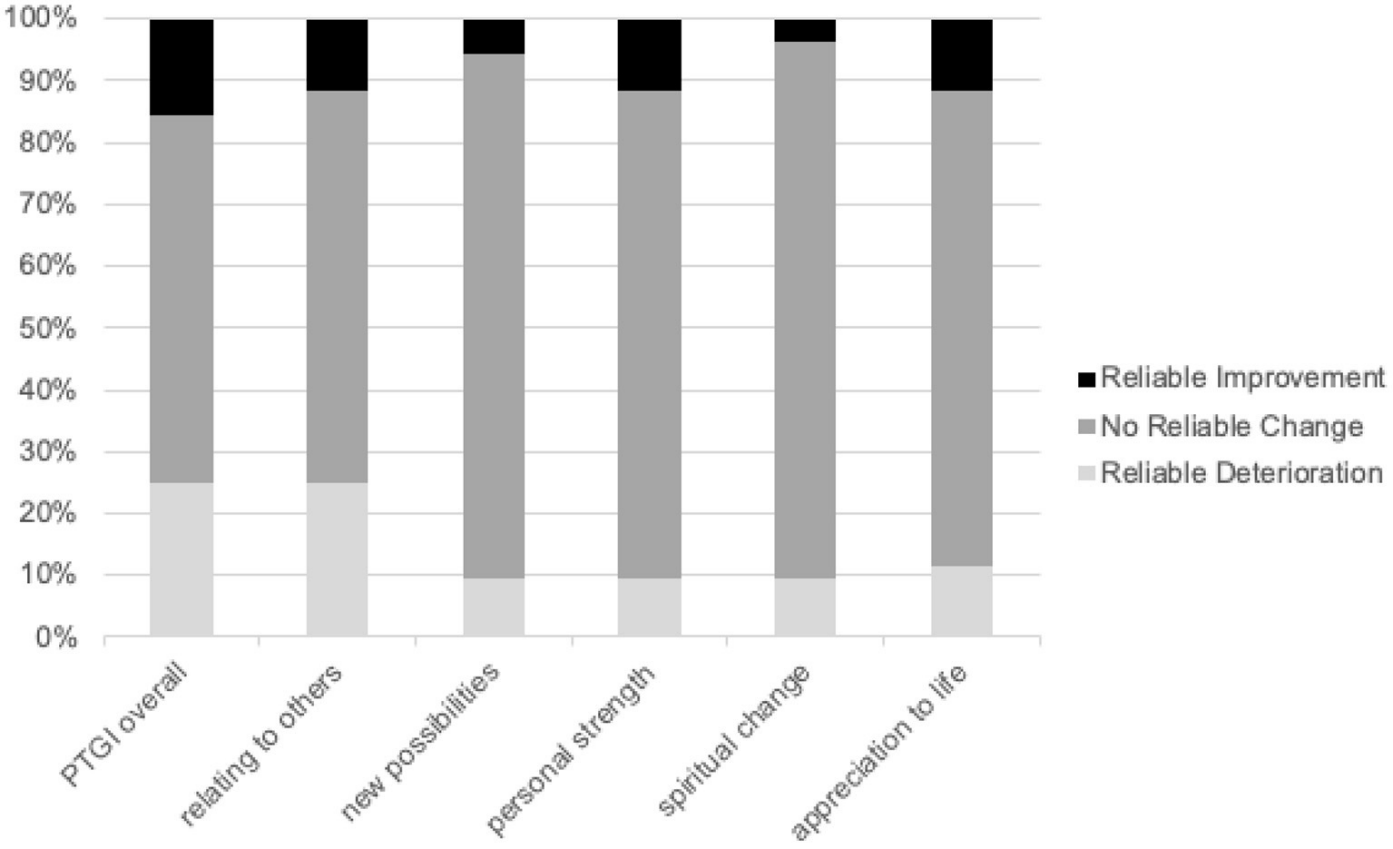
Reliable Change Index (RCI) of PTGI scores over a period of 1 year.

Figure 2 charts the reliable change index (RCI) of PTGI scores of respondents over a period of 1 year, with overall and domain scores represented. Most of the scores of the respondents were stable and did not report a reliable change in their PTGI overall scores (59.6%), relating to others domain (63.5%), new possibilities domain (84.6%), personal strength domain (78.9%), spiritual change domain (86.5%), and appreciation for life domain (76.9%).

Table 3 shows the repeated measures correlation of these constructs with PTGI total scores. Personal recovery and resilience correlated significantly with PTGI scores over time, while self-stigma, PANSS, employment and DUP did not. Supplementary Table 1 shows the sociodemographic correlates of PTG. Only the single/never married group were more likely to report PTG compared to their married counterparts (β = 16.30, *p* < 0.05).

**Table 3:** Factors associated with PTGI scores in respondents.

	**T1**		**T2**		**Correlation with total** **posttraumatic growth**	
	**Mean/n**	**SD/%**	**Mean/n**	**SD/%**	***r***	***p***
Personal recovery (QPR)	42.67	10.80	41.33	12.43	0.45	0.001
Resilience (CD-RISC-10)	22.65	10.06	22.79	8.15	0.47	0.000
Self-stigma (ISMI)	2.04	0.50	2.01	0.54	−0.14	0.326
**Positive and negative symptoms**						
PANSS positive	1.40	0.61	1.60	0.57	0.35	0.137
PANSS negative	1.48	0.65	1.55	0.62	−0.10	0.697
PANSS disorganized/concrete	1.27	0.51	1.38	0.52	0.11	0.654
PANSS excited	1.16	0.37	1.29	0.42	0.18	0.449
PANSS depressed	1.44	0.64	1.63	0.66	−0.12	0.625
Employment (employed)	39	40.6(%)	23	44.2(%)	0.22	0.117
Duration of Untreated Psychosis (DUP)	12.35	24.12	-	0.11	0.460	

## Discussion

In sum, we reported that PTGI is a reliable scale for measuring PTG in a sample of first episode psychosis patients seeking treatment from a specialized early psychosis intervention team in Singapore. We observed that PTG is present in our sample population, like reports of PTG in similar populations in other regions. PTGI scores remained stable over a period of 1 year against the backdrop of the Covid-19 pandemic in the sample population. Resilience and personal recovery were associated with PTG in our sample, while clinical indicators like symptoms severity (measured by PANSS and DUP) were not associated with PTG.

It is evident that PTGI is a relevant scale to chart the growth and development of people with first episode psychosis in the Singaporean context. This is aligned with the findings from an early psychosis unit based in the Canada (11) and to some extent qualitative findings from a series of semi-structured interviews performed in service users in the UK (12). Like our Canadian counterparts, most of the respondents in the study reported moderate to very great change for the better in various domains of their lives. Despite geographical and cultural differences between the Singaporean and Canadian contexts, very similar rates of endorsement of the various domains of PTGI were observed between the two sample populations, with the highest rates of endorsement for appreciation for life and lowest for spiritual change (11). Singapore is a secular city-state in Southeast Asia; it is not surprising that spiritual change is the least endorsed domain in our sample population. Furthermore, in recent years, more Singaporeans reported having no religious affiliations, particularly within the younger age groups (34, 35).

While Frazier et al. (20) did not question the existence of PTG in groups that experienced trauma, they were critical of PTGI, which they argued only captured “perceived” growth and not “actual” PTG of respondents. In their study, they had undergraduate students fill out scales which measured perceived PTG (using PTGI) and “actual” PTG (using a variety of scales like Ryff's positive relationship, meaning and satisfaction in life, gratitude questionnaire, and religious commitment inventory). Their findings suggested that PTGI was unable to detect growth after trauma in their sample population. Unlike the findings from Frazier et al. (20), we found convergence between the measures of actual PTG and perceived PTG in our sample group, which lends support to the construct validity of the PTGI scale. Even though Frazier and colleagues raised valid or relevant points about the PTGI being a scale that measures perceive growth retrospectively, we found it to be a valid, and robust scale for the measure of PTG in our sample population. A major difference between Frazier's and our work is the sample population. In this study, PTG was measured in a clinical population that had experienced first episode psychosis, a significant trauma, compared to Frazier's sample population in a group of undergraduate students in four universities in the United States. While Frazier et al. had pointed out that their population was at the peak age for trauma exposure, it may not be to the same degree as people who experience first episode psychosis. This finding is in line with the hypothesis put forth by Tedeschi and colleagues that PTG may only happen in people who experience trauma of “seismic” proportions (36).

This study is one of the first that followed the extent of PTG over an extended period. PTG was measured when the participants had received services from EPIP for 10–14 months and measured again when they were 21–27 months into the program. PTG was found to be a stable construct that did not change significantly over that one-year period between the two points of measure. Coincidentally, the data collection for the second timepoint overlapped with the period when Singapore was hit severely by the Covid-19 pandemic (April to May 2020). Despite multiple perspective pieces that warned of the mental health fallout in pandemic and post-pandemic world (37–39), it was observed that PTG remained stable in the respondents, and many did not show significant reliable decrease in their PTGI scores during the pandemic. The data suggests the growth obtained by the respondents were stable and did not change with the uncertainties brought about by the Covid-19 pandemic. In fact, a small, but vocal, group of lived experience researchers proposed that persons with mental health issues have much to share in coping with seismic life events, like a pandemic, because they already have been through one when they first encountered mental illness (40). They argued a case for the role of lived experience mental health leadership in the age of Covid-19. People living with severe mental health conditions could have wisdom to offer in the face of tremendous uncertainties and dealing with significant anxiety.

One of the aims of this study was to identify factors associated with PTG in the sample population. It was observed that resilience and personal recovery were associated with PTG. Recently, there is burgeoning evidence that people living with schizophrenia recover and even thrive in the aftermath of this severe mental illness (41, 42). Multiple psychosocial functioning and clinical factors were found to be associated and predicted by resilience (43, 44). A common thread that ran through these studies is that resilience is a protective factor in the course of mental illness. A few qualitative studies that delved into the process of recovery and resilience in young persons with first episode psychosis identified internal and external resources as key factors to the development of resilience in this group of people (45–47). Taken together with the data from this paper, it suggests that resilience can be cultivated, and it can lead to growth in the aftermath of the trauma of psychosis. Promoting resilience when working with people with first episode psychosis may go a long way in the development of PTG in this clinical population.

Increasingly, evidence from the literature shows that clinical recovery and personal recovery from severe mental illness are distinct constructs (41, 42, 48). Clinical recovery is typically defined in terms of symptoms remission and functional recovery, while personal recovery is a state of wellbeing aligned with the values of the person in recovery (49). The data from our study supports this notion, as we found that personal recovery is associated with PTG, while clinical measures of symptom severity like DUP and PANSS were not. The data from this study suggests that clinical recovery does not determine the extent of PTG in our sample population. The implication of this finding is significant as it suggests that the development of PTG does not depend on the severity of positive and negative symptoms. It does not matter how severe one's psychotic symptoms are; there is still a propensity for growth under the right support and environment around a person with first episode psychosis.

Having said that, growth in the aftermath of trauma, like early psychosis, is not a given. Despite most respondents reporting growth, there was still a subgroup that did not report growth and some even showed reliable decrease in their PTGI scores. Tedeschi and Calhoun (50), Janoff-Bulman (51), and Pietruch and Jobson (52) proposed that a combination of cognitive rumination, psychological preparedness, and self-disclosure were key factors in the development of PTG in the aftermath of trauma. Future studies could further explore key factors in the development of PTG post-psychosis.

There are several limitations to this study. Firstly, for a robust longitudinal study, the study design needs to follow up with its respondents for at least at three timepoints (53). However, due to the limitation of funding and resources, we were only able to follow up with a subset of the participants (*n* = 52) for one year at two timepoints. There is hence a limit to the analysis and conclusions we could draw from this data. In addition, there is a follow-up rate of 75%, which is not ideal since loss in follow-up is rarely random (54). Future studies could include a qualitative perspective on PTG to understand the mechanisms and individuals behind the phenomenon of PTG in this clinical population.

## Conclusion

Evidence from this study shows that outcomes of experiencing first episode psychosis may not be all distress and debilitation. Despite the severity of symptoms and the daunting challenges ahead of a young person who experienced first episode psychosis, there is a possibility of growth and development in the aftermath of this seismic event in that individual's life. It was observed that resilience and personal recovery were associated with PTG in the sample population. This has implications for clinicians working with people with first episode psychosis. There is a need to be aware that there is potential for this clinical population to recover and grow. The view on the outcomes of psychosis needs to be broadened to include the possibility of growth.

Recently, Ng and colleagues identified seven facilitators of PTG in psychosis through a systematic review. Summarized in the acronym, PROSPER, the facilitators were Personal identity and strength, Receiving support, Opportunities and possibilities, Strategies for coping, Perspective shift, Emotional experience, and Relationships (16). It is crucial for clinicians to be aware of the phenomenon of PTG in persons with psychosis to promote and facilitate its development. Growth after psychosis does not occur by chance. It must be cultivated and fostered through intentional conversations to bring about positive cognitive ruminations and self-disclosure. A qualitative study on EPIP patients' perspective of recovery revealed three areas in which were facilitators to personal recovery: personal agency, social and emotional support, treatment and services, faith and spirituality, and others resources (55). Encouraging a young person with FEP to take ownership of life and mental well-being could go a long way to enhance their recovery. Coupled with social support, treatment and other resources, a person with FEP may be able to recovery and grow in the aftermath of psychosis. Furthermore, there is a small, but growing, number of peer support specialists in Singapore (56). These mental healthcare workers could play a unique role in cultivating PTG in persons with first episode psychosis (16). Hearing about the peer support specialists' own narratives of recovery and growth could be the starting point for many persons living with psychosis to begin their journeys to recovery and growth (57).

## Data Availability Statement

The raw data supporting the conclusions of this article will be made available by the authors, without undue reservation.

## Ethics Statement

The studies involving human participants were reviewed and approved by National Healthcare Group Domain Specific Review Board. The patients/participants provided their written informed consent to participate in this study.

## Author Contributions

YL conceptualized the study, recruited participants, and wrote the first draft of the manuscript. VS recruited participants and ran the data analysis. YC recruited participants. SKV and MS helped with the conceptualization of the study. All authors gave intellectual inputs to the manuscript.

## Funding

This work was partially funded by a Psychosis Research Fund from Duke-NUS Medical School, Singapore.

## Conflict of Interest

The authors declare that the research was conducted in the absence of any commercial or financial relationships that could be construed as a potential conflict of interest.

## Publisher's Note

All claims expressed in this article are solely those of the authors and do not necessarily represent those of their affiliated organizations, or those of the publisher, the editors and the reviewers. Any product that may be evaluated in this article, or claim that may be made by its manufacturer, is not guaranteed or endorsed by the publisher.
